# Ivermectin to reduce malaria transmission II. Considerations regarding clinical development pathway

**DOI:** 10.1186/s12936-017-1802-3

**Published:** 2017-04-24

**Authors:** Carlos Chaccour, N. Regina Rabinovich

**Affiliations:** 1 ISGlobal, Barcelona Ctr. Int. Health Res. (CRESIB), Hospital Clínic, Universitat de Barcelona, Barcelona, Spain; 2 Centro de Investigação em Saúde de Manhiça, Maputo, Mozambique; 3 Instituto de Salud Tropical Universidad de Navarra, Pamplona, Spain; 4 Harvard T.H. Chan School of Public Health, Boston, MA USA

**Keywords:** Ivermectin, Endectocide, Clinical trials, Clinical development, *Anopheles*

## Abstract

The development of ivermectin as a complementary vector control tool will require good quality evidence. This paper reviews the different eco-epidemiological contexts in which mass drug administration with ivermectin could be useful. Potential scenarios and pharmacological strategies are compared in order to help guide trial design. The rationale for a particular timing of an ivermectin-based tool and some potentially useful outcome measures are suggested.

## Background

Vector control with long-lasting insecticidal nets (LLINs) or indoor residual spraying (IRS) is the one of major underlying reasons for the decline in malaria prevalence seen in the last 15 years [1]. Yet although currently available vector control tools can strongly reduce malaria transmission, reaching and sustain cero transmission is unlikely without innovation [2]; particularly in presence of Insecticide resistance [3] and residual malaria transmission [4].

Endectocides are systemic drugs that kill blood-feeding arthropods as well as internal parasites. They have been used in the veterinary market for more than 30 years. Of these, ivermectin was the first-in-class drug [5]. Since the 1980’s, ivermectin is used in humans for the treatment of onchocerciasis and lymphatic filariasis. Mass-administration of ivermectin could complement vector control with LLINs and IRS by reaching vectors that bite in unprotected temporal/spatial gaps or are resistant to insecticides.

As a potential new paradigm, early development should be guided by the results of semi-field or small-scale trials that could justify investment in large-scale field trials [6]. This paper provides a comprehensive assessment of the concepts that can influence the design of studies evaluating this potential new tool.

### A not-so-trivial question: transmission-blocking or vector control?

The term transmission-blocking drug refers to drugs that impede the transmission of the malaria parasite from humans to mosquitoes by killing gametocytes or inhibit the development of sporozoites in the mosquito [7]. Low-dose primaquine is recommended by World Health Organization (WHO) to all patients with parasitologically-confirmed *Plasmodium falciparum* malaria in order to block transmission from infected humans to mosquitoes [8]. Although there is guidance for potential development [9], there is currently no molecule in use to specifically inhibit parasite development in the mosquito.

If used at the appropriate dose and spacing, mass drug administration (MDA) with ivermectin could reduce malaria transmission, due mainly from the death of mosquitoes that feed on treated subjects [10, 11]. Additional benefit could result from reduced mosquito fitness and fertility [12–14], a shift in the mosquito population age structure towards younger females [11] and possibly, to a considerably lesser extent, by partial sporogony inhibition [15, 16] and a potential inhibition of hepatic schizonts [17]. Yet the impact on vectorial capacity would be driven mainly by a reduction of the daily probability of mosquito survival [18] as it is with LLINs and IRS, with the additional advantage of targeting the mosquitoes that bite outside protected environments and times (see “Residual transmission” below).

Ivermectin MDA would constitute a new paradigm for vector control and reducing transmission according to the criteria of the Vector Control Advisory Group (VCAG) on new tools [19] as it would:

(i) Offer indirect human protection by reducing local transmission (like indoor residual spraying does).
(ii) Have activity against different species of mosquitoes.
(iii) Work in the context of insecticide resistance, since the mechanism of action is different (glutamate-gated chlorine channels).
(iv) And cannot be described appropriately by an existing target product profile.

### Defining the ideal context for ivermectin use for malaria control

Ivermectin is not envisioned as a stand-alone tool. Any ivermectin-based intervention should be tested and deployed in conjunction with other WHO-recommended malaria control measures, including effective case management, vector control measures and drug-based prophylactic schemes in settings and groups applicable, such as SMC and IPTp. Ultimately, additional studies will be required to streamline the malaria control toolbox.

Four situations are defined for which an ivermectin-based tool could be a particularly valuable addition to current interventions.

#### Residual transmission

Residual transmission (RT) is defined as the transmission that persists after universal coverage with effective LLINs and/or IRS to which the local vectors are fully susceptible [4, 20–22]. It is the consequence of mosquito behaviour that defines the limits of what is achievable with these interventions and includes outdoor and early biting, outdoor resting, behavioural avoidance and feeding upon animals, as well as human behavioural factors (failure to utilize LLINs, outdoor sleeping). In this context, prolonged and appropriate coverage with LLINs/IRS can drive transmission to very low levels, but it is unlikely to interrupt transmission in some settings as the proportion of residual likely to increase progressively. RT is deemed as a significant challenge to malaria elimination which requires new or improved vector control methods or systems [4]. The development of new tools to address RT has been recognized as a priority by the WHO Global Malaria Programme [21].

Ivermectin distributed at population level would target mosquitoes feeding on treated subjects, regardless of the place or time of the blood meal, effectively targeting residual transmission.

Of note, one of the behavioural adaptations leading to residual transmission is a shift to feeding on animals [4, 20]; this could have implications for the use of ivermectin at the community level because untreated peridomestic animals would allow for this escape mechanism [23, 24], even after high human coverage. Ivermectin treatment of significant primary blood sources available to mosquitoes should be considered [25].

**Fig. 1:**
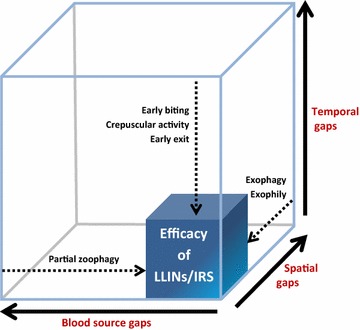
Temporal, spatial and blood-source gaps as a cause of residual malaria transmission. The gaps are not at scale. New interventions are needed to cover these gaps

Figure 1 is a schematic illustration (not at scale) of the temporal, spatial and blood sources gaps typically left uncovered by LLINs and IRS as a source of residual transmission.

#### Insecticide resistance

In 2012, when the Global Plan for Insecticide Resistance Management in malaria vectors was launched, resistant *Anopheles* had been identified in 64 malaria endemic countries, representing all WHO regions [26]. Of particular concern was the presence of resistance to all classes of insecticides in some areas and the appearance of high intensity of resistance up to 1000-fold known levels [27]. The presence of resistance to pyrethroids, the only insecticide used in LLINs, particularly in sub-Saharan Africa has worsened over the last 3 years [28], although the public health impact is still being debated [29]. Insecticide resistance is considered an important challenge for the sustainment of the achievements in malaria control over the last decade [1]. In this context, new strategies with novel mechanism of actions may be particularly valuable.

Ivermectin’s mechanism of action differs from all four classes of insecticides used today. Furthermore, a study done with *Anopheles coluzzi* carrying the *kdr* mutation associated with pyrethroid resistance showed they remained susceptible to ivermectin [30].

Ivermectin itself is not exempt of the theoretical possibility of resistance and should it be deployed for malaria vector control, there will be a need to monitor for the eventual appearance of resistance. Different isoforms of the glutamate-gated chlorine channels have been described in *Anopheles gambiae*, at least one of them is insensitive to the drug. The selective over-expression of this isoform could be a mechanism for ivermectin resistance [31]. To date, there are no reports of this occurring in the field. Additionally, ivermectin is a substrate of cytochrome P_450_ 3A4 [32] and to efflux pumps such as the P-glycoprotein [33], in the absence of fitness cost, overexpression of these enzymes/proteins could theoretically offer protection from ivermectin.

There is need for studies assessing the potential synergism of sub-lethal ivermectin doses on the susceptibility of mosquitoes to public health insecticides. This is based on proven reduced fitness after exposure to sub-lethal ivermectin doses [12, 34].

#### High transmission settings

The high vectorial capacity observed in sub-Saharan Africa has been identified as an important challenge to elimination [2, 35]. A baseline of high transmission decreases the technical and operational feasibility to achieve and maintain elimination, ultimately affecting the financial feasibility [36]. New paradigms in vector controls are needed to achieve and sustain markedly reduced transmission on the path to elimination in said areas.

#### Elimination settings

Any ivermectin based tool is expected to have a relatively short effect (weeks, rather than months or years), even with the hypothetical use of long-lasting formulations. Therefore, ivermectin’s profile fits well in the context of intense, time-limited efforts aimed at elimination. This could also limit the risk of resistance to the molecule.

#### Additional factors

Additional factors that could help select the most appropriate setting for proof-of-concept studies include:

- The presence of artemisinin resistance

The threat of artemisinin resistance spreading out of the greater Mekong subregion has created a sense of urgency given the current lack of other anti-malarials with the same efficacy and safety as artemisinin. The outdoor-biting behaviour of the local vectors makes ivermectin an attractive additional tool for local elimination.

- The specific susceptibility of the local vectors to ivermectin

Preliminary data suggest that different species of malaria vectors can have different susceptibility to ivermectin [37]. The dominant species in particular regions and their sensitivity to the drug should be taken into account when defining the target dose and scheme.

- The local transmission pattern

The local seasonality can greatly influence the efficacy of pulsed interventions on the basic reproductive number such as IRS or MDA with ACTs (this is partly dependent on the duration of effect) [38]. The main question deriving from this point is: when would be the best time to use/test an ivermectin-based tool? A partial answer is given below. Modelling is a key tool in the formulation of testable hypotheses in the context of other available data.

### Selecting the right proof-of-concept scenario: where is it testable?

The following concepts apply to ivermectin as a testable proof-of-concept requiring a change of label; for any other candidate endectocides the regulatory framework would need licensure of a new product. Selecting the appropriate scenario to prove the concept is a key point and for this, baseline epidemiology and baseline transmission measures must be taken into account. High transmission settings could shorten testing time while low transmission settings can be a financial challenge for initial studies due to larger sample size; but the implication of each will be different (reducing transmission vs driving to zero). Key outcome indicators should reflect transmission e.g. entomological inoculation rate (EIR) and/or incidence.

Six scenarios for the use of an ivermectin-based tool are proposed here using the criteria described above. In all cases, it is understood that ivermectin would be used as a complementary tool to core vector control measures and any other strategy used for an elimination/control campaign. All scenarios proposed here are envisaged as points in an elimination continuum and all assume an ivermectin campaign and community administration, regardless where in the continuum it is administered. Table 1 is just an example of how these scenarios could be ranked based on the criteria described in the previous section.

**Table 1:** This is an example of how the potential scenarios for a proof-of-concept study could be considered

	Residual transmission	Insecticide resistance	Transmission	Targeted for elimination	Artemisinin resistance	Burden of malaria	Testable
Elimination in the GMS	+++	+	+	+++	+++	+	Possibly
Elimination in selected areas of sub-Saharan Africa	++	+++	+++	+++	–	+++	Yes
Reduce disease burden in high-transmission areas	+	Any	+++	+		+++	Yes
Stem insecticide resistance in well-defined areas	Any	++++	Any	+	Any	+	Difficult
Elimination from hotspots in the endgame	++	+++	+++	+++	Any	+++ (local)	Doubtful
Stem of outbreaks	+	Any	++	Any	Any	+	Doubtful

Testable refers to the potential implications of the transmission pattern on sample size and the feasibility of controlled clinical trials for each particular scenario

*GMS* Greater Mekong Subregion

#### Elimination in the Greater Mekong Sub-region

The outdoor and early biting behaviour of the local vectors and the urgency created by artemisinin resistance support the use of novel tools in elimination efforts in this region. The local low levels of transmission raise the important question of whether a large enough sample size can be included to make the potential effect measurable.

#### Elimination in selected areas of countries with a heterogeneous transmission

In the face of high national burden of disease and threat of insecticide resistance, a complementary novel tool like ivermectin could help accelerate towards local [39] or sub-regional [40] malaria reduction or elimination targets.

#### Reduce disease burden in areas of high vectorial capacity

The effect of a single ivermectin intervention would only last for a few days to weeks depending on the dosing regimen and formulation used. This additional tool however, even if short-lasting, might serve as a complement to further reduce transmission and achieve consolidation in the context of elimination campaigns with multiple interventions.

#### Stem insecticide resistance in well-defined areas with high resistance intensity

In areas with resistance to multiple insecticides or with high insecticide resistance intensity, periodic deployment of an ivermectin-based tool could help suppress the resistant vector population while novel insecticides are introduced or elimination is assessed. This could be assessed by periodic evaluation of insecticide resistance markers. Of note, this would not be a regulatory endpoint for licensure.

#### Elimination from hotspots in the endgame

In the context of elimination efforts, modelling predicts that human-to-mosquito transmission efficiency will increase as malaria is controlled [41], also resistance may potentially concentrate in any hotspots left [42]. Ivermectin could be an additional tool in last mile focal efforts.

#### Control of outbreaks

Given its short duration, an ivermectin-based tool could prove useful when a quick, short-lasting suppression of the vectorial capacity is needed.

### Selecting ivermectin-based strategies for malaria control: factors affecting the potential impact

Ivermectin is expected to reduce the (EIR) by an amount that is influenced by:

#### The plasma levels reached as a factor of the LC_50_

The lethal concentration 50 (LC_50_) is the concentration of ivermectin in the imbibed solution or blood meal that kills 50% of the mosquitoes during a defined period of observation [43]. Although not usually reported that way, it should always include the timeframe i.e. 3-day-LC_50_ versus 10-day-LC_50_. The vector lethality caused by ivermectin is dose-dependent. As plasma levels increase and mosquitoes imbibe higher concentrations in bloodmeals, the time to reach 50% mosquito mortality will be shorter. Once plasma levels close to the 3-day-LC_99_ of a particular vector species are reached on an individual, almost all *Anopheles* from that species feeding on that particular individual will die before completing the gonotrophic cycle. This effect will be seen as long as said levels are sustained.

#### The duration of effective mosquitocidal concentrations

The direct mosquitocidal effect can only take place as long as the drug is present in the blood at effective concentrations [44], for *Anopheles* the 10-day-LC_50_ ranges from as low as 6 ng/ml for *Anopheles gambiae* [45] to 36 ng/ml for *Anopheles darlingi* [46] or 47 ng/ml for *Anopheles aquasalis* [47] (the former two represent 5-day-LC_50_). The duration of the mosquitocidal concentration will be in close relationship with the particular ivermectin susceptibility of the local vectors, it is yet to be assessed whether this could be related to metabolic resistance to other insecticides.

The relationship between plasma levels above the killing threshold and duration is likely to be nonlinear and possibly related to the area under the pharmacokinetic curve, but additional modelling work will be needed to prove this. Both parameters will be in direct relationship with the dose per body weight used and the number/spacing of the doses. The proposed process to design a trial will be to first define the target values of these parameters using modelling in order to choose the dose per body weight and scheme to be tested.

#### The population coverage

Modelling shows that a mass screening and treatment (MSAT) approach to ivermectin would have little effect on transmission. The real value of this tool would be in the context of community-based treatment. Modelling also points to higher coverage as an important driver of the potential impact of an ivermectin-based tool [44]. In other words, even if an MSAT strategy is used to treat the parasite, ivermectin would only have a relevant effect if given to as many eligible individuals as possible, regardless of RDT results.

#### The proportion of vectors feeding on alternative blood sources

Primarily zoophilic mosquitoes can sustain endemic malaria transmission even if they feed only rarely upon humans [4]. Untreated peridomestic animals could theoretically sustain mosquito populations even when all humans are ivermectin-treated. This could be a reason to consider including cattle and other peridomestic animals in endectocide-based strategies [25, 48], but this would require field data, both on the importance of these animals as an important source of nutrition, as well as the impact of treating them with ivermectin.

#### The magnitude and duration of beneficial sublethal effects of the drug on the mosquitoes

Mosquitos feeding at sublethal ivermectin concentrations are likely to experience reduced fertility, to fall temporarily to the floor, unable to fly (knock down) and to show incoordination while flying, all of which can contribute to additional vector mortality and reducing transmission of vector-borne diseases [12–14]. The relevance of these effects for entomological or epidemiological impact is still unclear.

**Fig. 2:**
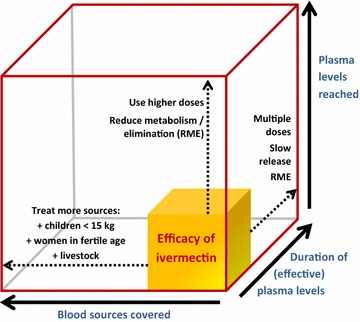
The theoretical efficacy of ivermectin mass drug administration based on three key parameters. Effective plasma levels would be directly linked to the specific ivermectin susceptibility of the local vectors. *RME* reduce metabolism or elimination e.g. using drugs

Figure 2 schematically illustrates how the concepts of plasma levels reached, duration of said levels and coverage of blood sources combine to determine the theoretical magnitude of effect of ivermectin MDA for malaria control.

### Selecting ivermectin-based strategies for malaria control: how to use it?

#### Using the current oral formulation

The current oral formulation is used for onchocerciasis at the 150–200 µg/kg/dose with a frequency of one to four times a year in different settings, but killing mosquitoes was not the intended outcome of this regimen, and until recently there were no data on the entomological impact of this large-scale use. There is now evidence that ivermectin MDA at this dose can reduce the 3-day survival of mosquitoes caught in the area for up to 1 week after MDA [49, 50]. This results in the age structure of the mosquito population being shifted towards younger, less infectious ages for up to 3 weeks and a significant reduction of the sporozoite rate to levels as low as 20% of the pre-MDA ones for 2 weeks [49, 51].

Considerations for using the current formulation at the onchocerciasis-approved doses should be based on the clear determination of the susceptibility (LC_50_) of the main local vector species and the modelled impact of different doses and schemes. The results of recently finished trials could help to further parametrize the models [52, 53]. Doses of 200 mcg/kg repeated every 3 weeks can have a measurable impact on malaria incidence but this implies a intense logistical efforts [54]. Data are emerging on a variety of options, and the final regimen will need to balance biological impact and operational feasibility.

Finally, the current oral formulation could be used, in a clinical trial context, at different doses and frequency to provide “proof of concept” that ivermectin delivered for a specified number of days via MDA program would have a measurable public health and mosquito outcome, this can guide the development of novel formulations.

#### Potential novel formulations

Modified formulations have been used in animal studies as a way to deliver stable mosquitocidal concentrations of ivermectin for longer periods of time (ranging from 7 to >30 ng/ml) [55–57] from 2 to 24 weeks. Using the existing formulation can prove the concept, but whether that multiple-dose regimen is deliverable at scale will define whether a new formulation with different performance characteristics would be optimal.

#### Target coverage

The target coverage will be defined with help from modelling; key points will be the exclusion of certain groups (children, pregnant or potentially pregnant women due to the lack of safety data of higher or more frequent doses) and the demographic characteristics of the population. The importance of alternative blood sources from peridomestic animals and their potential role in sustaining mosquito populations should be contemplated when defining the biological coverage of an ivermectin-based intervention [25, 48].

**Fig. 3:**
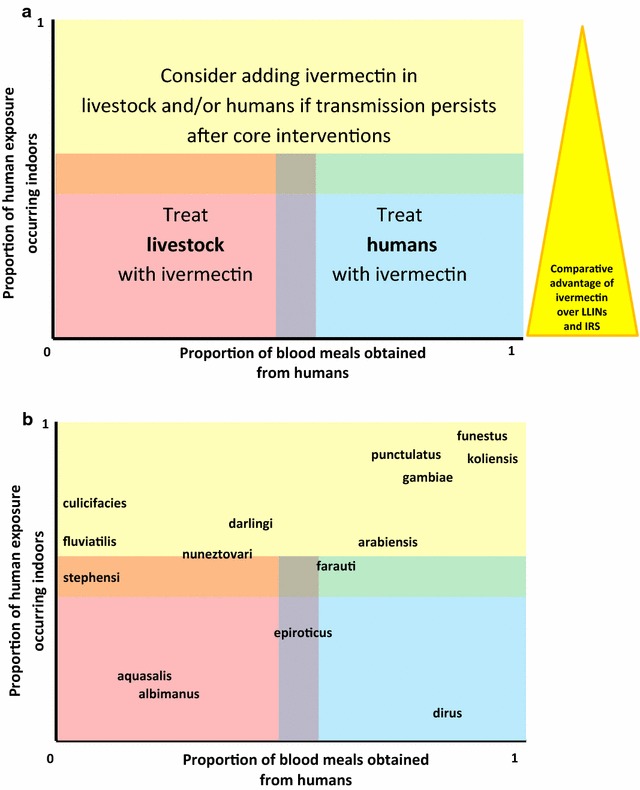
Theoretical scenarios for the use of ivermectin in humans and/or peri-domestic animals according to behaviour of dominant vectors. In **a** the *coloured squares* are used for illustration purposes as there are no clear limits for these scenarios. There are no “pure” scenarios in which mosquitoes bite only humans outdoors, so ivermectin should always be envisaged as a complementary measure. **b** shows how ivermectin use in a specific setting could be tailored according to the behaviour of the main vectors. It has been adapted with permission from Killeen et al. [58]

Panel A in Fig. 3 illustrates the different scenarios in which ivermectin could be used in animals or peri-domestic animals according to vector behaviour as well as the potential comparative advantage of ivermectin over LLINs and IRS in some settings, panel B is adapted with permission from Killeen et al. [58] and shows how ivermectin use could be tailored to the humans or livestock in different areas after the behaviour of the main local vectors.

#### Examples of possible use

The possibilities include using the current oral formulation at different doses and spacing or developing a novel long-lasting formulation. In all cases, the use of ivermectin would be in addition to core vector control tools with or without MDA to reduce the parasite pool at population level.

##### Using the current oral formulation at high doses for a short period of time

In this design, ivermectin is distributed at high doses (that is six to nine-fold the total dose approved for onchocerciasis distributed across several days) in order to increase the peak concentration in plasma and consequently the time above mosquitocidal concentrations.

##### Using the current oral formulation at Onchocerca-approved doses *at intervals*

In this design, ivermectin is distributed at usual doses for a long time (200 mcg/kg every 3 weeks for months) to suppress the vector population or at intervals aimed at modifying the age structure of the local vector populations.

##### Novel, slow release formulations of ivermectin

Developing novel, long-lasting formulations can increase the duration of the mosquitocidal effect after a single encounter [55, 56]. Injectable formulations seem to be the easier solution but could challenge implementation. Transdermal formulations may be acceptable to the population but require additional time and investment in R&D. The recently described ultra-slow release oral formulation by Bellinger et al. [56] is an elegant solution; it is capable to safely deliver mosquito-killing ivermectin concentrations for at least 2 weeks after a single dose and offers the possibility to combine several drug treatments at once [59].

The three envisioned possibilities are compared for potential advantages and disadvantages in Table 2. Of note, during clinical development it will be key to achieve equilibrium between efficacy (avoid too low doses) and safety (avoid too high doses).

**Table 2:** Ranking different potential designs for ivermectin-based tools

Regimen	Efficacy	Safety	Acceptability	Compliance	Programmatic difficulty	R&D costs	Implementation costs
High dose, single encounter	To be assessed	To be assessed	+	+++	+	+	Similar to ACT MDA
Existing dose, multiple encounters	To be assessed	+	+++	+	+++	+	Similar to SMC
Novel long-lasting formulation, single encounter	To be assessed	To be assessed	To be assessed	+++	+	+++	Similar to SMC but high R&D costs and longer timeframe to availability

*ACT* artemisinin combination therapies, *MDA* mass drug administration, *SMC* seasonal malaria chemoprophylaxis, *R&D* research and development

### Timing the intervention: when could ivermectin be most useful?

In many settings, malaria elimination programmes could resort to MDA campaigns to reduce the human reservoir [7, 60]. The timing of this intervention will be critical. It must be remembered however, that even after theoretical mass administration of these drugs with anticipated exclusions, a small but significant proportion of the parasite pool can survive in sporogonic stages in the mosquito [61]. Historically, the 1969–1976 Garki project pointed to the highest impact of MDA on transmission was seen during the dry season, when transmission was at its lowest and the parasite pool smaller [62] (this was based on parasite treatment, not endectocides).

**Fig. 4:**
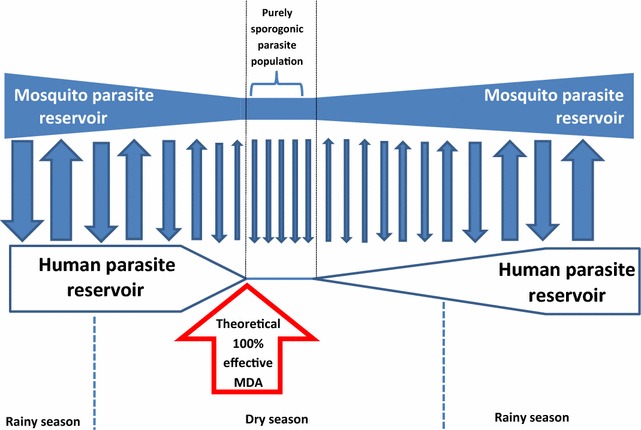
The transmission between mosquito and human parasite pools. The parasite reservoir in the mosquito could allow for transmission to continue even after a fully effective MDA campaign. Adapted from Killeen 2013 [61]. The best timing for deployment of an ivermectin-based tool would be right before the *red arrow* and in combination with other vector control measures. *MDA* mass drug administration

It has been suggested that even after well-timed MDA parasite treatment campaigns, the proportion of parasites in the sporogonic stage in the mosquitoes, safe from the effects of drugs, can allow for transmission to continue after the dry season [61]. Figure 4 illustrates this concept.

Following this reasoning, the best moment to implement an ivermectin-based strategy would be prior to any parasite-reducing MDA; this could help reduce the mosquito to human transmission responsible for the “leap-frog” pattern of transmission after MDA [61]. Additional modelling is needed in this aspect with the results of data of timing under various conditions. Final decisions on timing will need to take into account operational feasibility related to potential co-administrations and the influence of seasonality in accessing the communities.

### Study design for a proof-of concept of ivermectin MDA to achieve a measurable transmission reduction

#### Potential outcomes

Ivermectin MDA would be a transmission-reduction tool. As such, the best outcome measures would be directly related to transmission in humans and mosquitoes [63, 64]. The beneficial effect would be measured at community level. Outcome measures could be divided in epidemiological, entomological and laboratory-based. Efficacy measured through human endpoints will depend on baseline transmission intensity, requires robust baseline data and is likely to vary across different transmission settings. The primary endpoint should show a tangible benefit for the population. The WHO-recommended primary efficacy endpoint for phases IIb and III of malaria vaccine trials is incidence of all episodes of malaria [65]. Case definition and case detection method must be clearly defined [65].

For transmission-blocking vaccines, it is suggested that entomological endpoints should fall into the category of secondary or exploratory endpoints [64]. However, in the case of ivermectin, the main effect is a reduction of transmission achieved by killing an important proportion of the vector population, hence entomological endpoints need to be included among the primary outcome measures. Of note, EIR and related metrics are hard to measure reliably, operator dependent and can have substantial variation [66], alternative entomological endpoints could encompass variations on the mosquito population age structure and residual transmission. These are unlikely to suffice regulatory requirements but could be key intervening variables to explain the impact (or lack thereof) of the trial results.

Table 3 shows some examples of potential primary outcomes measures for clinical trials of ivermectin. See Tusting et al. [63] or Pinder et al. [64] for comprehensive reviews on measures of malaria transmission.

**Table 3:** Potential primary outcome measures for clinical trials of an ivermectin-based vector control tool

	Outcome measure	Rationale	Method	Advantages	Disadvantages
In Humans	Clinical incidence	Of primary importance for the target population	Clinical case definition and laboratory confirmation	Unequivocal and tangible reflection of benefit for the population The earliest measurable clinical end-point reflecting transmission reduction	Requires robust baseline data Must reflect seasonal variations
	Parasite prevalence	Directly related to the EIR and VC	Consensus needed Options include: microscopy RDT PCR RT-PCR QT-NASBA [75] LAMP [76]	Robust measure Used across many settings Tangible reflection of benefit at population level	Laborious. Requires robust baseline data Must reflect seasonal variations Some methods are not suitable for the field. Method must be tailored according to the local prevalence
	Gametocytaemia	A measure of infectiousness to mosquitoes (k) [63]	Consensus needed Options include: RT-PCR (RNA vs DNA) LAMP QT-NASBA	Robust measure of transmission	Must collect baseline data May not reflect population benefit May not reflect the independent effect of ivermectin
	Variations in population level serology [77]	Indirect measure of transmission	Consensus needed on mosquito and parasite antigens	Direct reflection of exposure to malaria vectors and parasites Most useful in very low-transmission settings Useful without baseline data	May not reflect clinical benefit Must reflect seasonal variations
	Molecular force of infection [78]	Indirect measure of transmission	PCR	Reliable and easier to determine than FOI	May not reflect clinical benefit Seasonality Inter-cluster variations
Entomological	Entomological inoculation rate [66]	A direct measure of transmission intensity. Likely to reflect the additional effect of ivermectin	Human landing catches vs light traps for biting rate Dissection, ELISA or PCR for sporozoite rate	Most useful in high transmission settings	Requires extensive knowledge of the local vectors. Very laborious. Minimum EIR of 5-10 needed for reliability [64] Ethics of human landing catches Must avoid contamination from control sites
	Vectorial capacity	Would additionally reflect the daily survival of vectors, a more direct effect of ivermectin	As above plus determination of the daily survival and assessment of the extrinsic incubation period	The most direct assessment of transmission	Requires extensive knowledge of the local vectors. Very laborious. Minimum EIR of 5-10 needed for reliability [64] Ethics of human landing catches Must avoid contamination from control sites

*EIR* entomological inoculation rate, *FOI* force of infection, *LAMP* loop-mediated isothermal amplification, *PCR* polymerase chain reaction, *QTNASBA* real-time quantitative nucleic acid sequence-based amplification, *RDT* rapid diagnostic test, *RT-PCR* real-time PCR

An important secondary analysis would be the effect of ivermectin on prevalence and intensity of NTDs and ectoparasites. In areas of co-endemicity, capturing these and other co-morbidities can help analyse the true impact of this potential tool.

Additional potential secondary outcomes include: the safety profile of the ivermectin regime, malaria incidence and transmission on the following season, cost-effectiveness and community perception. A decision regarding endpoints needs to be made in consultation with regulatory agencies and informed by WHO.

#### Potential comparators

Any ivermectin-based tool needs to be used in an MDA campaign. The main question is: *does ivermectin add value to existing intervention packages?* this could be seen in terms of transmission, time to impact, costs or effectiveness.

##### *Community standard core vector control interventions alone* (LLINs/IRS vs LLINs/IRS + ivermectin)

Using core vector control measures as comparator, without directly targeting the parasite with MDA would allow direct measurement of the impact of ivermectin in the presence of other vector control tools. This is likely to be needed whether ivermectin is envisaged as a target product or target partner drug for MDA. It may also have the benefit of the simplest study design and cost. This would be the simplest way of capturing the added value of ivermectin as a vector intervention and the referent for primary regulatory endpoint, to be discussed with regulatory agencies.

##### *MDA with anti*-*malarials* (ACT MDA+LLINs+/−IRS vs ACT MDA +LLINs/+/−IRS + ivermectin)

Current elimination strategies use ACTs that quickly clear parasitaemia, provide prophylactic effect and reduce carriage of immature gametocytes [67]. These campaigns are accompanied by vector control interventions as well as robust surveillance for case detection and treatment. Using these interventions as comparator with appropriate power would allow for the determination of any additional benefit provided by a systemic insecticide like ivermectin to the leading hypothesis for accelerating elimination, particularly in Africa.

##### *Transmission*-*blocking interventions*

The only intervention currently available is the use of primaquine to clear gametocytes. The effect of primaquine is primarily reducing the infectiousness of humans to mosquitoes. Ivermectin would primarily reduce vector density. Although both ultimately reduce transmission, a direct comparison would fail to acknowledge their very different mechanism of action and potential synergistic effect. Therefore, this is not the best approach, particularly for a regulatory endpoint.

#### Potential trial design

An individually-randomized clinical trial would fail to measure the expected community effect. Definitive proof of efficacy will arise from community or cluster randomized trials. While there have to be enough clusters to meet tests for robustness given hypothesized effect size, and the specifics of the control intervention package may vary, there are, conceptually, some key elements to consider:

- A vector control package representative of strategies and epidemiology for that region must be included.
- Appropriate surveillance system for identification of cases and appropriate response systems must be in place. This includes reporting systems to ensure timely facility-based reporting.
- The impact of ivermectin MDA is modelled to be proportionally higher in areas of high transmission [44, 56], giving a theoretical power advantage to that context, although trials under different scenarios (higher endemicity to accelerate the path to elimination and at low levels of endemicity to accelerate crashing transmission) have been considered and would be valuable in different context.

Critical to trial design is the primary endpoint—as discussed above. Under the specific scenario of high endemicity, elimination strategy, cluster randomized MDA ivermectin, an illustrative endpoint would be the community impact (public health), with key secondary endpoints (transmission; cases).

Detailed discussion of trial design is beyond the scope of this document, the reader is referred to recent comprehensive reviews on the design of trials to assess vector control and transmission blocking tools [68, 69]. There are also potential ethical implications of trials assessing a drug that reduces transmission but does not provide a direct individual benefit. These issues has been particularly discusses in the context of transmission-blocking vaccines [70] and are reviewed with a focus on endectocides in the third paper of this thematic series [71].

#### Go/No-Go criteria for pre-clinical and early clinical development

As a reference point, the initially proposed parameters for transmission blocking vaccines included proportion of oocyst reduction in a proportion of vaccinees for a defined period of time, i.e. >50% reduction in oocyst count in >50% of the vaccinees to advance from phase Ia to Ib and >80% reduction in oocyst count in >80% of the vaccinees for 9 months to advance from a Ib trial [64]. PATH’s original 2010 TPP for a transmission blocking vaccine proposed an 85% transmission-blocking efficacy as the efficacy target. Total oocyst prevalence has also been proposed as a more suitable reflection of infectivity [72]. Finally in laboratory populations, even modest reduction in vertebrate-to-insect transmission of 32% can eliminate *Plasmodium* infections [73].

For an ivermectin-based tool, early entomological Go/No-Go criteria could include:

- Cumulative 3-day mosquito mortality: reflecting a quick reduction in vector densities and with direct implication on an effect on human-to-mosquito transmission.
- Cumulative 9-day mosquito mortality (before completion of sporogony): reflecting the reduction in infectious vectors.
- The duration of the above effects.
- Definitive proof of impact on human health will likely be required at later development stages given its importance for communities and policy makers.

The fact that sublethal concentrations can also reduce transmission by impairing flying and fertility should be taken into account as the total effect in the field could be higher than the expected effect just based on mortality. These could be assessed by evaluating the behaviour of vectors caught alive by using exit traps or equivalent methods. Decisions need to be made on (a) the appropriate measurements of impact on human health, (b) measurements that can help understand how the effect was reached (and what would need to be optimized in future studies) and that could in the future be considered as trial endpoints and (c) measurements that would lead to future hypotheses.

## Conclusions

The clinical development of any ivermectin-based tool intended to reduce malaria transmission will require at least one pivotal cluster-randomized trial. For the planning of such a trial two key points must be considered, the eco-epidemiological scenario in which the trial will take place and the way ivermectin will be administered to the population. Mainly because of sample size, it is likely that running such a trial in an area of high seasonal transmission would have the best value for early investment.

## Abbreviations

ACT: artemisinin-based combination therapy
EIR: entomological inoculation rate
FOI: force of infection
GMS: Greater Mekong Subregion
IRS: indoor residual spraying
LC_50_: lethal concentration 50
LF: lymphatic filariasis
LAMP: loop-mediated isothermal amplification
LLINs: long-lasting insecticide-treated nets
MDA: mass drug administration
NTDs: neglected tropical diseases
PCR: polymerase chain reaction
QTNASBA: real-time quantitative nucleic acid sequence-based amplification
RDT: rapid diagnostic test
RT-PCR: real-time PCR
